# Annotations

**Published:** 1890-12-27

**Authors:** 


### Annotations.

**Diphtheria at Marlborough School.**

It is satisfactory to learn on authority that there have been no new cases of diphtheria at Marlborough School during the last few weeks. We trust, however, that the observations made in our issue of November 30th will be laid to heart by the responsible authorities at Marlborough and other public schools. There is some fear, however, that, in consequence of the light and airy manner in which Dr. Louis Parkes seems disposed to regard the outbreak, it may be treated as a kind of accidental and inexplicable occurrence, and that no really practical steps will be taken to prevent with certain similar outbreaks in the future. Dr. Louis Parkes, we under stand, is of opinion that the criticism of the editor of The Hospital is made without any knowledge of the facts, and is wide of the mark. He has no doubt in his own mind about the origin of the diphtheria, and he can "say positively that there is no danger to any boy now in the school." Concerning which, the first criticism that will occur to any man of experience is, that Dr. Louis Parkes must be an exceeding bold and rash person to "say positively" anything of the kind. We dare lay a very considerable wager that if he lives and practises medicine for twenty or thirty years longer, he will at least learn to speak with caution, and to avoid giving assurances of safety in cases where no such thing as certainty is by any means possible. Moreover, so far from our criticism being made without an adequate knowledge of the facts, we had all the circumstances as plainly put before us as headmasters' logic and printers' ink could put them. And still further, the writer of the article was a physician of large experience in diphtheria, both in private practice and at public schools. In expressing the opinion that the original boy who took the disease, or at any rate first showed it at the school, and was sent home and cured, and three weeks afterwards returned to school and was passed as quite well both by the doctor at home and the school doctor—in expressing the opinion that this boy's second attack was caused by a poison at school, and that the other boy who was attacked almost simultaneously owed his seizure also to a school poison, the writer of the article gave a judgment which experience warrants, and which the large majority of competent physicians would have concurred in. But it is bootless to wrangle over questions of this kind. The point is, have the school authorities made the school sanitation absolutely perfect? If they have they will probably have no more diphtheria, either from without or from within. If, on the authority of Dr. Louis Parkes, or for the sake of saving a few pounds, they have stopped short at doing everything that science and thoroughness demanded, on them will lie the responsibility of future outbreaks, which may bring untold anguish to parents and injury to the school.

**Wholesome Agnosticism Wanted.**

"The world, as it exists for the student of physical science, is a purely mechanical system. All physical process as such is ultimately reducible to the motion of matter in space. This view has been extended so as to apply even to the voluntary actions of human beings. From the purely physical point of view consciousness counts for nothing as a real factor in cosmic process. The writing of *Hamlet* was a result determined purely by a mechanical process in which certain molecular changes in Shakespeare's brain played a dominant part." So writes a reviewer in the *Speaker*, without so far as appears, altogether agreeing with the views of those who regard the world as a purely mechanical system. The probability is that the reviewer is not a physiologist, and has never had a very intimate acquaintance with physiologists and physiological chemists. If he had known them as well as some of us know them he would not have been so inclined to take their views *au sérieux*. The plain and simple truth is that the physiologist and the physiological chemist know nothing whatever about the *modus operandi* of thought production. They know that a man has a brain; and that sensation travels inward and registers itself there; and that motion is originated and travels outwards. But the physiological laboratory tells us no more about thought production than the stars tell us about their inhabitants. All this the physiologists themselves know perfectly well; and yet they stand by their hypotheses as if they were as demonstrable as propositions in Euclid. They should say they do not know. They are quite prompt and ready with a declaration of agnosticism when the question of the being and nature of God, or of the possibility of a revelation is broached. Then "the only attitude possible for a scientific man is one of agnosticism" they tell us. "They do not know, they cannot know, they stand in mute awe before the tremendous mystery of the unknowable." Quite so, and we should sympathise with them much more sincerely if they would extend their agnosticism into other regions of the unknowable. Why one man is a Shakespeare, another a Bacon, and a third a Henry the Eighth, what physiologist can tell us? And why Shakespeare, born and bred as he was, could write "Hamlet," whilst thousands of public schoolboys every year scarcely succeed in taking poll degrees, which of them knows? We are all getting a little weary of the vast amount of "original research" that leads to nothing. Metaphysicians for ages have been wasting the world's time in threshing over again the empty straw of metaphysical speculation. Men hoped for better things of the physiologists. They ought, at least, to tell us when they have come to the end of their tether, and when they cannot see a single inch further into the mysteries of man and of being. Instead of that, no sooner do they come into complete darkness, than they begin guessing and quarrelling, exactly as the metaphysicians did before them. This is not science, but only a modern method of wasting time. No physiologist knows anything more of the kind of molecular changes that took place in Shakespeare's brain when he wrote "Hamlet" than Shakespeare himself knew.

**Ill-health and Crippled Powers.**

"Ill-health, we believe, has much to do with the crippling of his powers." This is a kindly critic's explanation and apology for what he feels bound to describe as a less interesting and powerful story than readers had a right to expect from a certain well-known author. Volumes of truthful commonplaces might be written on such a text. Authors and other hard-worked men hate commonplaces. But *Ex nihilo nihil fit*, which, being liberally interpreted, means that you cannot have in a book what the author has not in his head; nor can you have successful energies expended in business which the business man has not in his nervous system. Is there any real literary or business worker in London of the age of forty who has not found out that the want of health, of physical and mental force and facility, builds more brick walls across the pathway of advancement than any other single cause whatsoever? What wonders some of us could do if the energy were equal to the will! But it is not. A man has to learn how much he can do every day, and do well. When he has learnt that he will be very wise if he does not attempt more. By keeping himself a little within the limits of what he can do well, he will gradually acquire the capacity to do a little more; and conversely, by daily doing a little more than he has energy for, he will as surely acquire a capacity for doing a good deal less, and for doing it badly. Hurry is the murderer of excellence. It is doubtful if any man ever gained immortality who was always in a hurry, or ever will. Who expects to gain immortality? Only a few, perhaps! But every man of even moderate sense and judgment, both expects and wishes to do the best he can for those who depend upon him, and for his own name and fame. He never can do anything like his best if he is always in a fuss and a fume. Fuss and fume destroy health; and, as a general rule, the measure of health is, and ought to be, the measure of the excellence of work. If a man be not a fool he understands his physical and mental possibilities at forty; if he be not a still greater fool, he measures his work by his capabilities. Of course there are certain forms of ill-health that are unavoidable. Of those we do not here speak. We only desire to insist that ill-health cripples the powers with the utmost sureness; and that those who desire, as all men should desire, always to be doing better and better work, must resolutely look to their health, and learn to make it and keep it good. "Town" and "strain" can only be endured as the result of training. A certain number of men kill their bodies in the training, and many more kill their minds. Intellectual men are often as stupid and as wilfully ignorant of this sort of learning as the unintelligent masses are of literature. Literary men frequently despise doctors and their ways. That is a mark of foolishness. Ignorance of health questions, and indifference thereto, bring revenges of a kind that the intellectual man finds all but intolerable.

